# KDM4B and KDM4A promote endometrial cancer progression by regulating androgen receptor, c-myc, and p27^kip1^

**DOI:** 10.18632/oncotarget.5165

**Published:** 2015-09-11

**Authors:** Mei-Ting Qiu, Qiong Fan, Zhu Zhu, Suet-Ying Kwan, Limo Chen, Jin-Hong Chen, Zuo-Lin Ying, Ye Zhou, Wei Gu, Li-Hua Wang, Wei-Wei Cheng, Jianfang Zeng, Xiao-Ping Wan, Samuel C. Mok, Kwong-Kwok Wong, Wei Bao

**Affiliations:** ^1^ Departments of Obstetrics and Gynecology, International Peace Maternity & Child Health Hospital affiliated with the Shanghai Jiao Tong University School of Medicine, Shanghai, China; ^2^ Department of Gynecologic Oncology and Reproductive Medicine, The University of Texas MD Anderson Cancer Center, Houston, TX, USA; ^3^ Department of Thoracic/Head and Neck Medical Oncology, The University of Texas MD Anderson Cancer Center, Houston, TX, USA; ^4^ Departments of Obstetrics and Gynecology, Shanghai First Maternity and Infant Hospital, TongJi University School of Medicine, Shanghai, China; ^5^ Department of Dermatology, Shanghai First People's Hospital Affiliated to Shanghai Jiao Tong University School of Medicine, Shanghai, China; ^6^ Department of Laboratory Medicine and the Center for Stem Cell and Developmental Biology, The University of Texas MD Anderson Cancer Center, Houston, TX, USA

**Keywords:** endometrial cancer, lysine demethylases KDM4B and KDM4A, androgen receptor, histone modification, c-myc, p27^kip1^, prognosis

## Abstract

Epidemiological evidence suggests that elevated androgen levels and genetic variation related to the androgen receptor (AR) increase the risk of endometrial cancer (EC). However, the role of AR in EC is poorly understood. We report that two members of the histone demethylase KDM4 family act as major regulators of AR transcriptional activityin EC. In the MFE-296 cell line, KDM4B and AR upregulate c-myc expression, while in AN3CA cells KDM4A and AR downregulate p27^kip1^. Additionally, KDM4B expression is positively correlated with AR expression in EC cell lines with high baseline AR expression, while KDM4A and AR expression are positively correlated in low-AR cell lines. In clinical specimens, both KDM4B and KDM4A expression are significantly higher in EC tissues than that in normal endometrium. Finally, patients with alterations in AR, KDM4B, KDM4A, and c-myc have poor overall and disease-free survival rates. Together, these findings demonstrate that KDM4B and KDM4A promote EC progression by regulating AR activity.

## INTRODUCTION

Endometrial cancer (EC) is the most common gynecological malignancy. In the United States alone, 54, 870 new EC diagnoses and 10, 170 fatalities are expected in 2015. Hysterectomy with or without adjuvant treatment for localized EC yields a 5-year survival rate of approximately 95%. However, treatment outcome is poor in the 28% of EC cases that are diagnosed in an advanced stage [1]. Dysregulation of steroid hormones plays a major role in EC pathology, and hormone therapy is a common component of treatment for advanced EC. 33% of patients respond to hormone therapy, which currently targets mainly estrogen and progestogens [2]. Because it is fertility-sparing, hormone therapy has also been a mainstay of EC treatments in young women; however, the overall response rate in that demographic is only 68% [3].

Recent research on the effects of androgens on endometrial tissue might have important implications for EC treatment. Polycystic ovarian syndrome (PCOS), an androgen-related disease characterized by prolonged periods of anovulation, has long been associated with increased risk of EC. PCOS and EC share many of the same risk factors, and androgens might also promote the development of EC tumors. A recent Australian study found that women with PCOS were at a four times higher risk of developing EC than women without PCOS. This risk of EC was associated with symptoms of androgen excess, such as hirsutism (odds ratio [OR] 2.4, all EC cases) and irregular periods (OR 3.1, all EC cases) [4], further indicating that high androgen levels may promote EC development.

The androgen receptor (AR), a nuclear transcription factor, regulates EC progression. AR can inhibit the growth of endometrial epithelial cells in culture, and this effect is blocked by an AR antagonist [5]. However, other findings suggest that AR may promote EC by increasing CD133 expression, cell migration, and epithelial-mesenchymal transition [6]. We have previously demonstrated that AR promotes EC cell proliferation via activation of the target gene c-myc [7]. However, the effects of AR activity on EC remain largely unknown.

Enzymes that regulate reversible histone lysine methylation also might play an important role in EC [8]. One such family of enzymes is the KDM4 demethylases, which includes KDM4A-D (previously called JMJD2A-D). KDM4 enzymes mainly recognize dimethylated and trimethylated histone H3 on lysine 9 (H3K9) to activate transcription. Dimethylated H3K36 and trimethylated H1.4K26 are also substrates for KDM4 enzymes [9]. Interestingly, KDM4 enzymes bind to AR to co-regulate transcription; depletion of any of the KDM4 proteins suppresses AR-mediated transcription *in vitro* and *in vivo* [10–12]. Finally, KDM4B, which itself is regulated by androgens, can stimulate AR-mediated transcription not only via demethylation activity but also via modulation of ubiquitination in prostate cancer cells [13]. These observations suggest that specific KDM4 family members might contribute to EC progression by modulating AR activity.

Here, we conducted a variety of *in vitro* and *in vivo* studies to identify the effects of KDM4 enzyme activity on AR signaling and EC progression. Levels of the four KDM4 proteins were decreased using siRNA in different cellular models of EC, and resulting changes in AR signaling and EC progression were measured using qRT-PCR, immunoassays, and measurements of cellular migration and proliferation. Additionally, known target genes of AR were probed in these cell lines to determine specific downstream molecular effects of manipulating KDM4 levels. Because KDM4 enzymes are important regulators of histone methylation, epigenetic changes were also examined in transfected cells. The use of cell lines with both high and low baseline AR expression AR allowed us to identify distinct roles for KDM4 proteins in EC. Xenograft experiments in which mice were injected with EC cells with either normal or reduced levels of specific KDM4 proteins confirmed their effects *in vivo*. Finally, tissues and genetic information from human patients with EC were examined to confirm the potential clinical relevance of our findings regarding KDM4 and AR.

## RESULTS

### *In silico* analysis of AR-KDM4B signaling in EC

**Figure 1:**
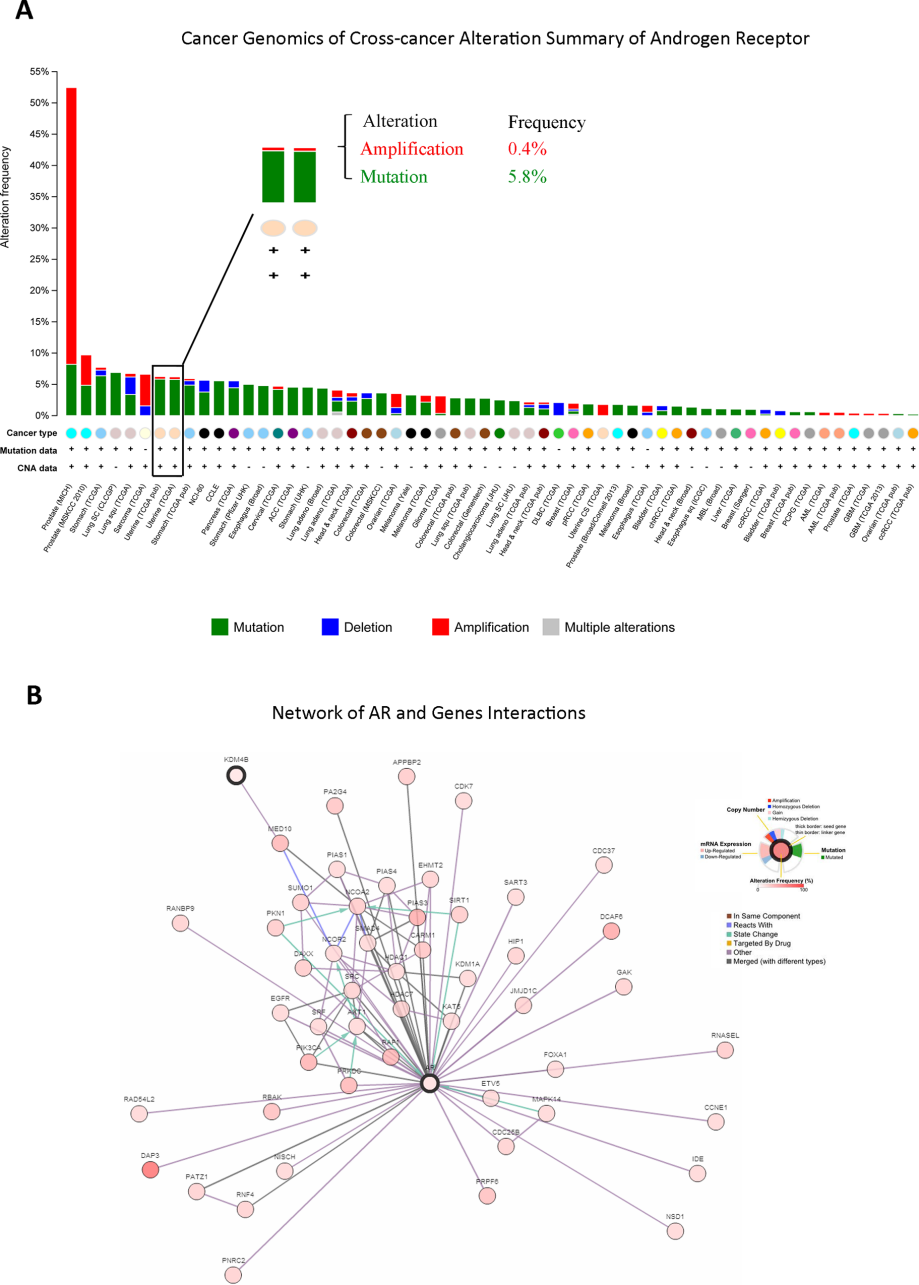
*In silico* analysis of patient database identified novel AR-KDM4B signaling in EC **A.** Genomics of cross-cancer alteration summary of AR in all cancers, which included AR amplification, mutation, and deletion. **B.** AR and relative gene interactions network using existing data from The Cancer Genome Atlas.

To help establish whether AR signaling affects EC progression, we used cBioPortal to examine cross-cancer alteration summaries of AR, which included AR amplification, mutation, and deletion (Figure 1A). EC patients had an AR alteration rate of more than 5%, including amplification (0.4%) and mutation (5.8%); EC ranked seventh out of all cancers in this regard (Figure 1A). We then assessed interactions between AR and other epigenetic regulators using existing data from TCGA to identify which regulators might affect EC progression (Figure 1B). This analysis pointed to a role for KDM4B in AR co-regulation and EC.

### KDM4B binds to AR and activates AR-mediated transcription in MFE-296 EC cells

**Figure 2:**
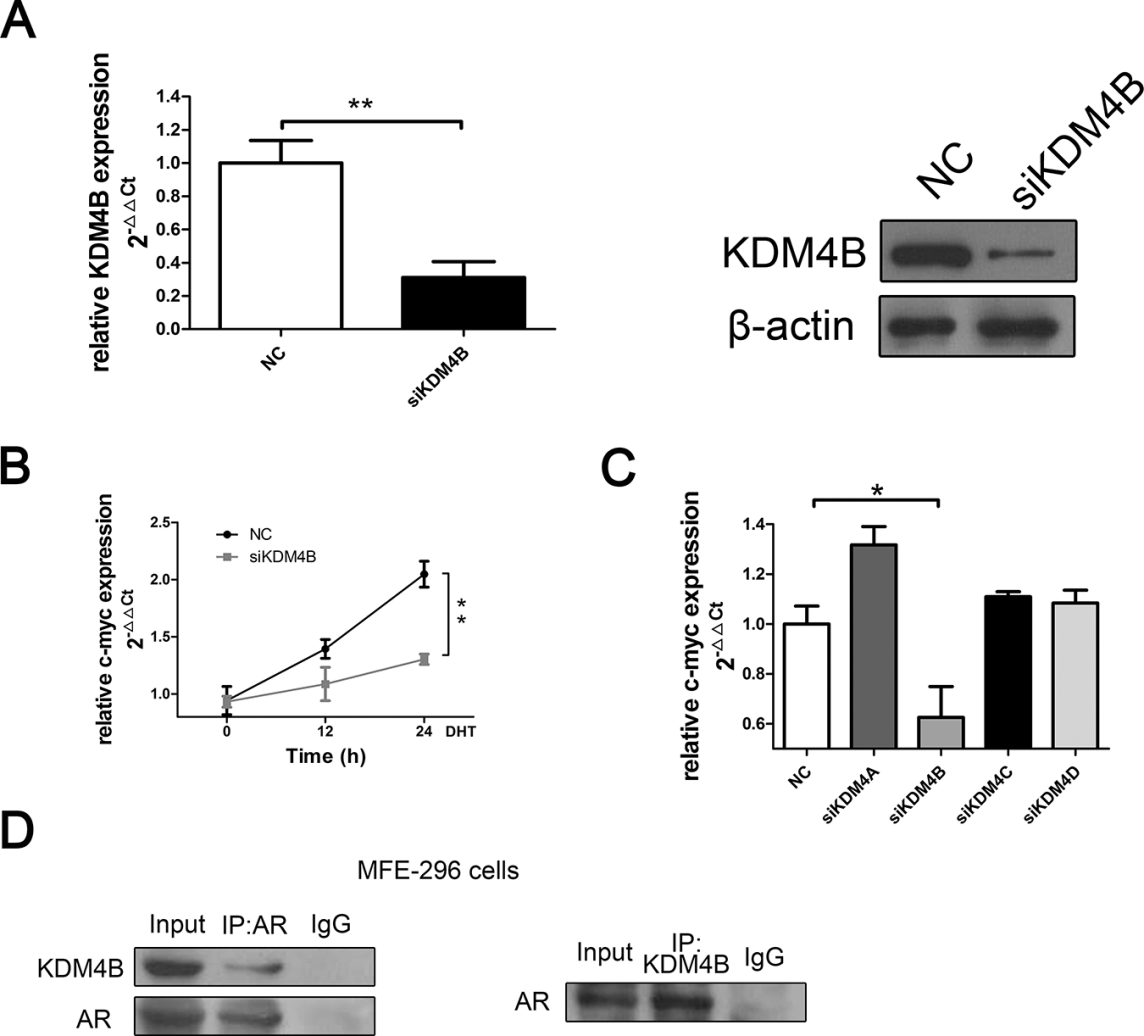
KDM4B binds with AR and activates AR-mediated transcription in MFE-296 EC cells **A.** KDM4B knockdown was confirmed by qRT-PCR and Western blotting in MFE-296 cells. β-actin was used as a loading control. **B.** MFE-296 cells were transiently transfected with either negative control (NC) or KDM4B (siKDM4B) siRNA in steroid-depleted media and treated with 100 nM DHT for up to 24 h before RNA extraction. qRT-PCR was used to assess c-myc mRNA expression. **C.** KDM4B silencing inhibited c-myc mRNA expression in MFE-296 cells, whereas knockdown of other KDM4 enzymes didn't affect c-myc expression. **D.** MFE-296 cells grown in serum-containing media were subject to co-IP using anti-AR, anti-KDM4B, or control antibodies before Western blotting using reciprocal antibodies. All experiments were performed two or more times, and data represent the mean fold change ± SE.

We used siRNAs to decrease expression of each KDM4 methylase in order to determine whether these proteins affect AR signaling and EC cells. RT-PCR revealed that depletion of KDM4B down-regulated expression of the AR-dependent gene c-myc (Figure 2A and 2B) in MFE-296 cells. This effect was specific to KDM4B; knock-down of other KDM4 family members did not affect c-myc expression (Figure 2C). Furthermore, qRT-PCR revealed that none of three non-KDM4 epigenetic regulators known to affect AR signaling (KDM1A, JMJD1C, and SMAD4) affected c-myc expression (Supplementary figure 1). Additionally, coimmunoprecipitation revealed that KDM4B binds to AR in MFE-296 cells (Figure 2D).

### KDM4B, cooperating with AR, promotes clonogenic growth, migration, and invasion of EC cells both *in vitro* and *in vivo*

**Figure 3:**
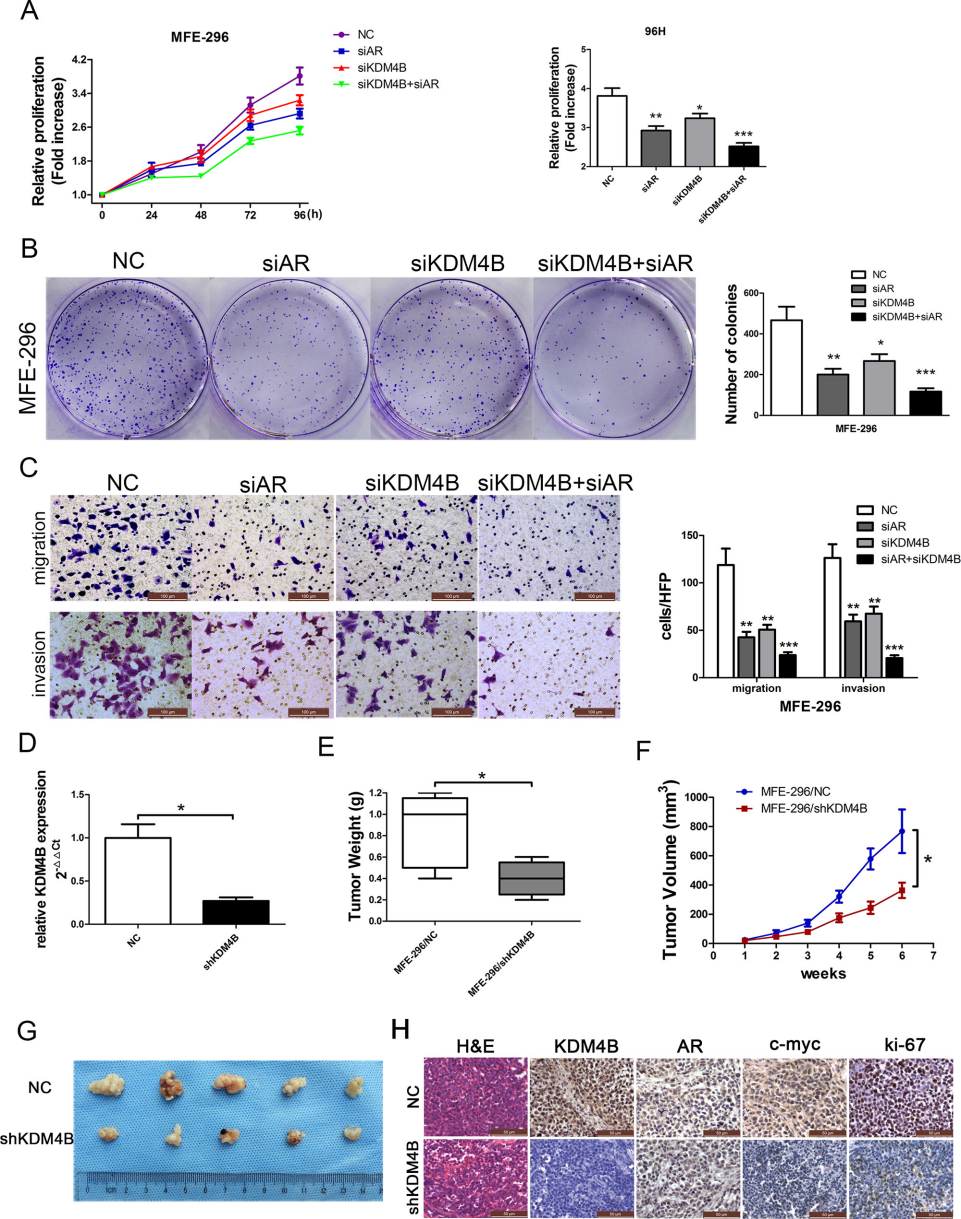
KDM4B promotes AR-mediated carcinogenesis in MFE-296 cells MFE-296 cells were transiently transfected with negative control (NC), AR (siAR) siRNA, KDM4B (siKDM4B) siRNA or KDM4B and AR siRNA together (siKDM4B+siAR). Cell proliferation was determined by MTT assay **A.** and colony formation assays **B, C.** Migrated and invasive MFE-296 cells on the lower surface of the Transwell filter were stained and photographed, 200×. The number of migrated and invasive cells is shown on the right. *P < 0.05, **P < 0.01, ***P < 0.005 compared with the NC group. **D.** KDM4B knockdown efficiency in shKDM4B group was confirmed by qRT-PCR. The tumor weight **E.** and tumor volumes **F.** and **G.** formed from nude mice injected subcutaneously with MFE-296 cells stably transfected with NC (MFE-296/NC) or shKDM4B (MFE-296/shKDM4B) were shown. **H.** Staining with hematoxylin and eosin (H&E) or immunohistochemical staining for KDM4B, AR, c-myc, and ki-67 in mouse tumor tissues (400×).

To test the effects of KDM4B knockdown on EC progression, MFE-296 cells were transfected with siRNA against AR, KDM4B, both AR and KDM4B, or a negative control (NC). Proliferation of MFE-296 cells decreased after knockdown of either AR or KDM4B. When both AR and KDM4B were knocked down, proliferation was further inhibited in MTT and clonogenic growth assays (Figure 3A and 3B). Moreover, when both AR and KDM4B were inhibited, the numbers of migrated and invasive cells were reduced (Figure 3C). We also confirmed that MFE-296 cells stably transfected with shKDM4B (MFE-296/shKDM4B) showed reduced expression of the protein as compared to cells transfected with negative control (MFE-296/NC) (Figure 3D).

Two groups of mice were injected with MFE-296/shKDM4B and MFE-296/NC cells, and tumors resulting from the xenograft were measured, to test for changes in EC progression *in vivo*. The final mean weight and volume of tumors in the MFE-296/shKDM4B group were significantly lower than those in the MFE-296/NC group (*P < 0.05; Figure 3E–3G). Xenograft immunohistochemistry revealed lower expression of KDM4B, c-myc, and ki67 (a common proliferation index) in the MFE-296/shKDM4B group compared to the control group (Figure 3H).

### In response to androgens, KDM4B activates AR target c-myc by demethylating H3K9me3 in MFE-296 cells

**Figure 4:**
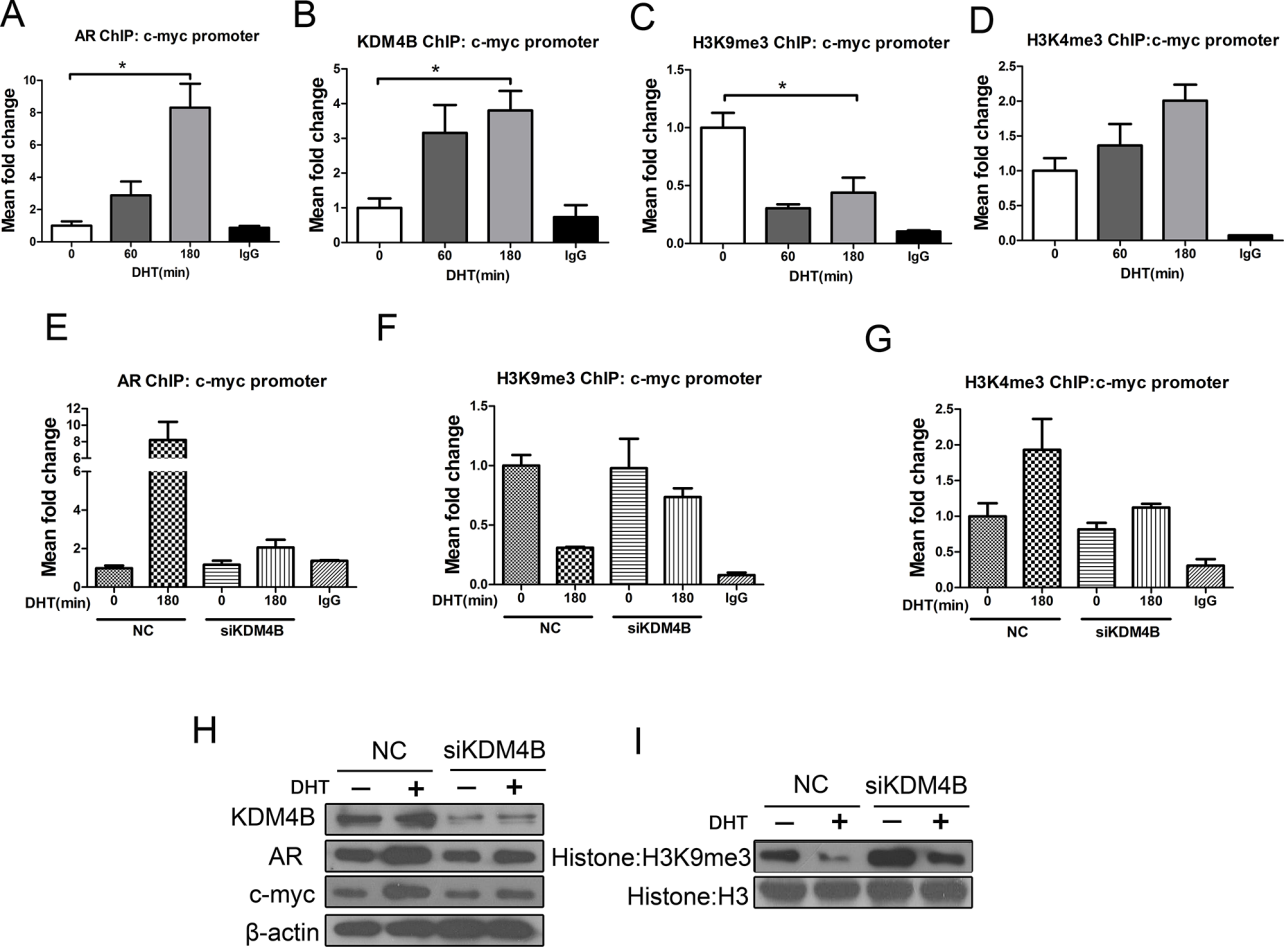
KDM4B activates AR target c-myc by demethylating H3K9me3 in response to androgens in MFE-296 cells A series of ChIP assays for **A.** AR, **B.** KDM4B, **C.** H3K9me3, and **D.** H3K4me3 at the c-myc promoter in MFE-296 cells treated with DHT (100 nM) for 0, 60, or 180 min. *P < 0.05 compared with 0 min. MFE-296 cells were transiently transfected with either negative control (NC) or KDM4B siRNA (siKDM4B) and placed in steroid-depleted media for 72 h with or without a 180 min exposure to 100 nM DHT. ChIP was then performed with an anti-AR antibody **E.** an anti-H3K9me3 antibody **F.** or an anti-H3K4me3 antibody **G.** and analyzed at the c-myc promoter. Nonspecific IgG was used as a negative control. **H.** MFE-296 cells were subject to transient transfection with either negative control or KDM4B (siKDM4B) siRNAs in steroid-depleted media for 48 h before a 24 h to 100nM DHT. After protein extraction, AR, KDM4B, c-myc (H), and global histone modifications **I.** were assessed by Western blotting. Histone H3 and β-actin were used as loading controls.

In MFE-296 cells, KDM4B and AR were recruited to the c-myc promoter and H3K9me3 methylation was reduced (Figure 4A–4C). Conversely, H3K4me3 methylation was enriched in response to DHT (Figure 4D). Depletion of KDM4B attenuated ligand-dependent recruitment of the AR (Figure 4E), increased H3K9me3 (Figure 4F), decreased H3K4me3 (Figure 4G), and reduced c-myc protein expression (Figure 4H). KDM4B knockdown also increased H3K9me3 methylation at the protein level (Figure 4I). Western blots confirmed that KDM4B expression in MFE-296 cells was higher than KDM4B in normal endometrial cells (Supplementary Figure 2A and 2B).

### In response to androgens, KDM4A binds to AR and suppresses AR target p27^kip1^ by demethylating H3K4me3 in AN3CA cells

**Figure 5:**
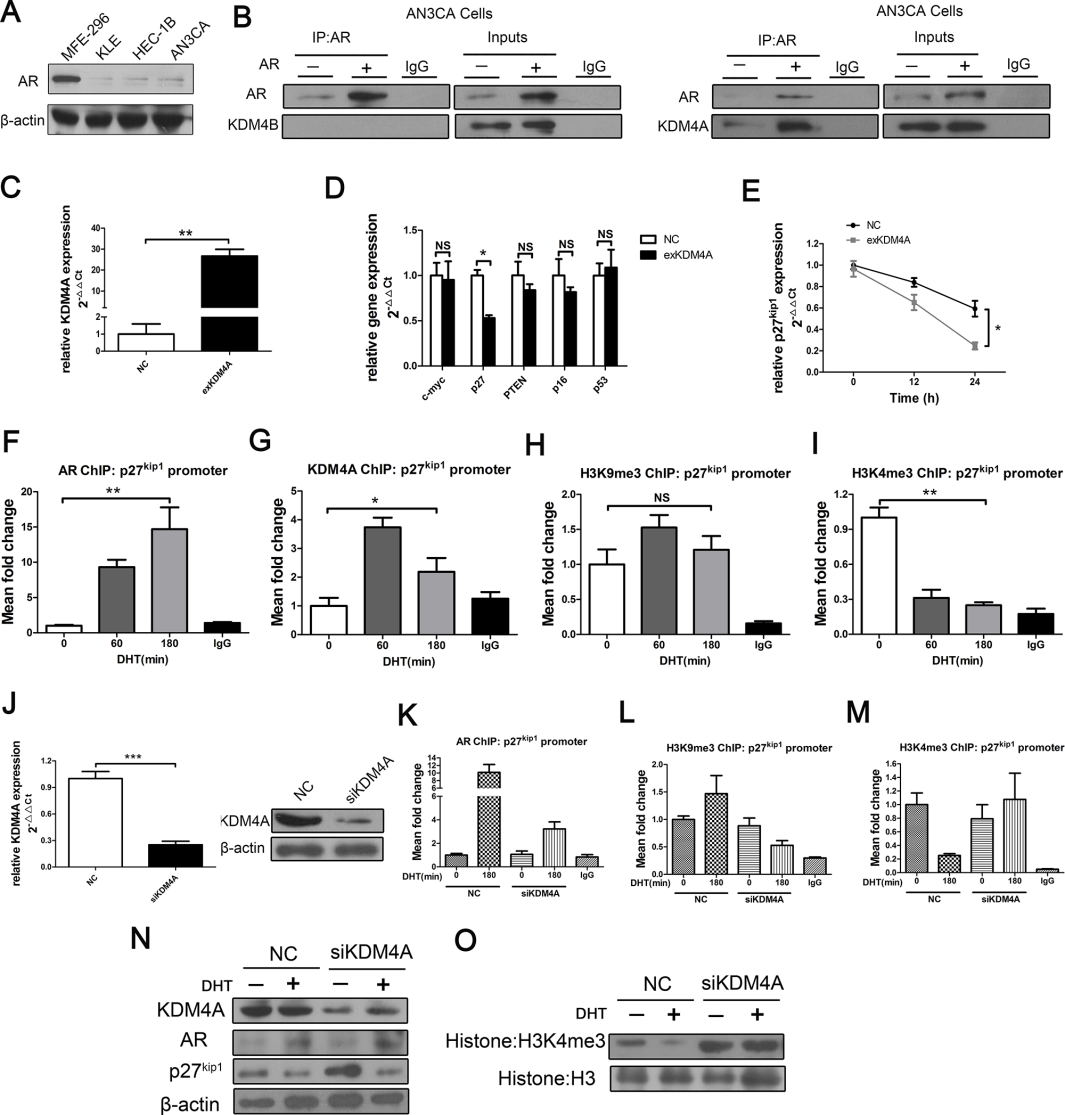
KDM4A, but not KDM4B, binds with AR and suppresses AR target p27^kip1^ by demethylating H3K4me3 in response to androgens in AN3CA cells **A.** AR expression in various EC cell lines; AN3CA cells were studied because of their low AR expression. **B.** AN3CA cells transiently transfected with mammalian expression vectors encoding AR or empty vector control were subject to co-IP using anti-AR or control antibodies before Western blotting using anti-KDM4B (B, left) or anti-KDM4A (B, right). **C.** KDM4A over-expression was confirmed by qRT-PCR in AN3CA cells. **D.** AN3CA cells were transiently transfected with either negative control (NC) or KDM4A expression plasmid (exKDM4A). Overexpression of KDM4A inhibited p27^kip1^ mRNA expression in qRT-PCR assays in AN3CA cells **E.** the expression of other suppressor genes, including c-myc, did not affect p27^kip1^ levels (D) *P < 0.05 compared with NC. ChIP for **F.** AR, **G.** KDM4A, **H.** H3K9me3, and **I.** H3K4me3 at the p27^kip1^ promoter in AN3CA cells treated with DHT (100 nM) for 0, 60, or 180 min. *P < 0.05, **P < 0.01 compared with 0 min. **J.** AN3CA cells were transiently transfected with either negative control (NC) or KDM4A siRNA (siKDM4A) in steroid-depleted media for 72 h with or without 180 min of exposure to 100 nM DHT before ChIP with an anti-AR antibody **K.** an anti-H3K9me3 antibody **L.** or an anti- H3K4me3 antibody **M.** and subsequently analyzed at the p27^kip1^ promoter. Nonspecific IgG was used as a negative control. **N.** AN3CA cells were subject to transient transfection with either negative control or KDM4A (siKDM4A) siRNAs in steroid-depleted media for 48 h before 24 h of exposure to 100nM DHT followed by protein extraction. AR, KDM4A, p27^kip1^ (N) and global histone modifications **O.** were assessed with Western blots. Histone H3 and β-actin were used as loading controls.

To determine whether KDM4B and AR interact in other EC cell lines, we performed co-immunoprecipitation (co-IP) in AN3CA cells, which have low levels of AR (Figure 5A). KDM4B did not co-precipitate with AR in either wild-type AN3CA cells or in AN3CA cells ectopically overexpressing AR (Figure 5B, left). However, KDM4A did co-precipitate with AR in AN3CA cells overexpressing AR (Figure 5B, right). KDM4C and KDM4D did not interact with AR (data not shown).

Overexpression of KDM4A did not affect c-myc expression in AN3CA cells (Figure 5C and 5D). We then analyzed the expression of several tumor suppressor genes (p27^kip1^/p16/PTEN/p53) [14–17] after AN3CA cells were transfected with KDM4A plasmid (exKDM4A) or NC (Figure 5C). KDM4A overexpression inhibited the expression of the AR-dependent p27^kip1^ gene (Figure 5D and 5E) in response to 100 nM DHT for 24 h in AN3CA cells; no significant changes were observed in p16, PTEN and p53 mRNA expression (Figure 5D).

In AN3CA cells, KDM4A was recruited to the p27^kip1^ promoter in response to androgens. DHT treatment also recruited AR to the p27^kip1^ promoter (Figure 5F–5G), reduced H3K4me3 methylation (Figure 5H–5I), and increased H3K9me3 methylation (Figure 5H). KDM4A knockdown attenuated ligand-dependent recruitment of the AR (Figure 5J and 5K), decreased H3K9me3 (Figure 5L), and increased H3K4me3 (Figure 5M), consistent with increased p27^kip1^ protein expression (Figure 5N). Inhibition of H3K4me3 due to KDM4A was also seen at the protein level and seemed to affect the whole histone 3 protein (Figure 5O). DHT treatment also resulted in the recruitment of KDM4A to the p27^kip1^ promoter in HEC-1B cells, another line with low AR levels. (Supplementary Figure 3A). Furthermore, KDM4A knockdown attenuated ligand-dependent recruitment of the AR and increased H3K4me3 methylation in these cells, consistent with increased p27^kip1^ expression (Supplementary Figure 3B–3D). The observed effects were specific to KDM4A; KDM5A, another known H3K4me3 demethylase [18], neither demethylated H3K4me3 nor inhibited p27^kip1^ expression in AN3CA cells. (Supplementary Figure 4A–4C).

### KDM4A, cooperating with AR, promotes clonogenic growth, migration, and invasion of EC cells both *in vitro* and *in vivo*

**Figure 6:**
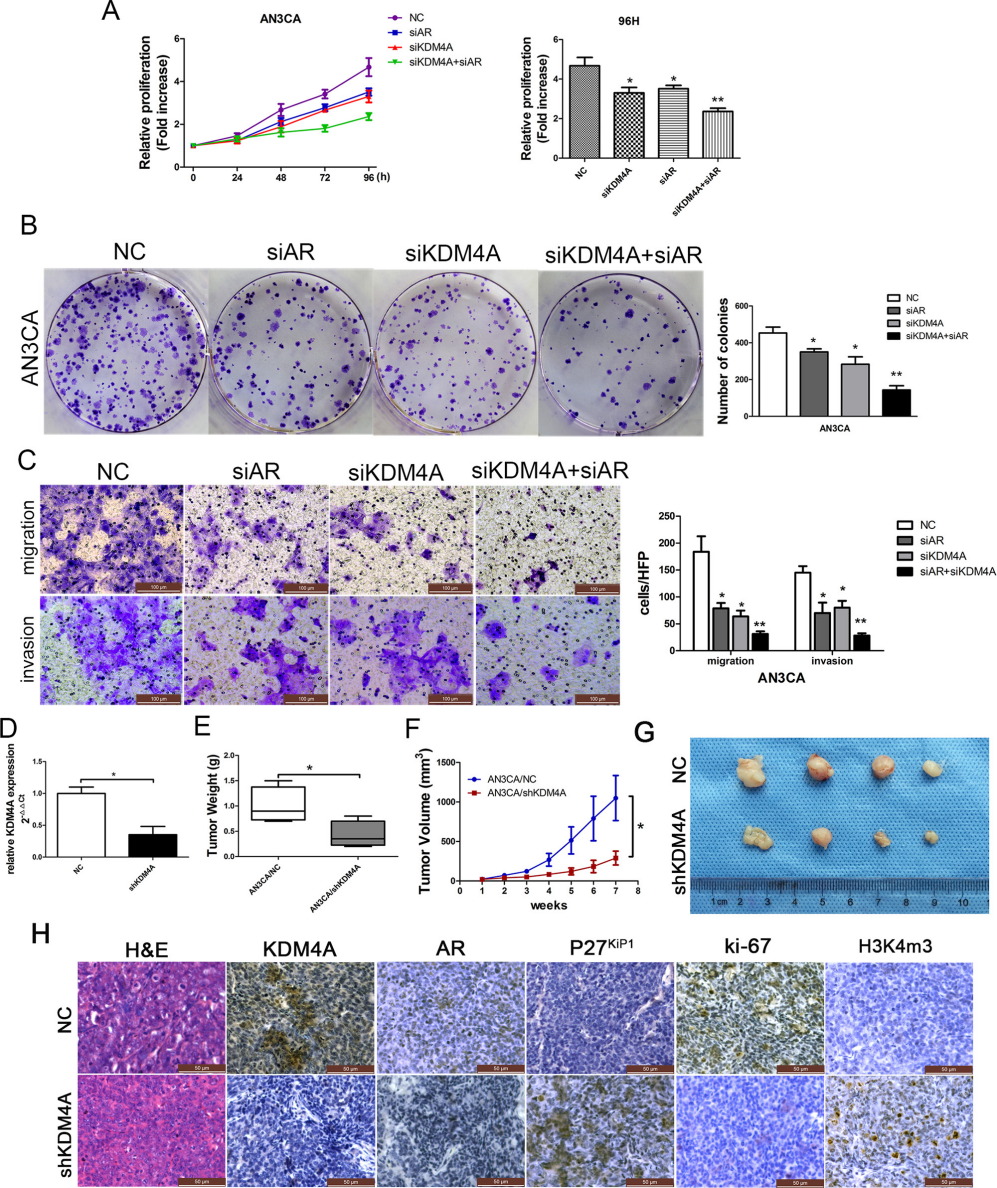
KDM4A, but not KDM4B, promotes AR-mediated carcinogenesis in AN3CA cells **A.** AN3CA cells were transiently transfected with negative control (NC), AR (siAR) siRNA, KDM4A (siKDM4A) siRNA or KDM4A and AR siRNA together (siKDM4A+siAR). Cell proliferation was determined by the MTT assay (A) and colony formation assays **B, C.** Migrated and invasive AN3CA cells on the lower surface of the Transwell filter were stained and photographed, 200×. The number of migrated and invasive cells is shown on the right. *P < 0.05, **P < 0.01 compared with the NC group. **D.** KDM4A knockdown efficiency in shKDM4A group was confirmed by qRT-PCR. Tumor weight **E.** and tumor volumes **F.** and **G.** from nude mice injected subcutaneously with AN3CA cells stably transfected with NC (AN3CA/NC) or shKDM4A (AN3CA/shKDM4A). **H.** Staining with hematoxylin and eosin (H&E) or immunohistochemical staining for KDM4A, AR, p27^kip1^, ki-67 and H3k4me3 in mouse tumor tissues (400×).

The effects of KDM4A and AR on EC progression in AN3CA cells were measured with proliferation assays. AN3CA cell proliferation was reduced by siAR or siKDM4A. When both AR and KDM4A were reduced, proliferation was further inhibited (Figure 6A and Figure 6B), and the numbers of migrated and invasive cells were also decreased (*P < 0.05, **P < 0.01; Figure 6C). Proliferation was also examined *in vivo* using tumor measurements from mice injected with AN3CA cells transfected with either shKDM4A (AN3CA/shKDM4A) or NC (AN3CA/NC) (Figure 5D). The final mean weight and volume of tumors in the AN3CA/shKDM4A group were significantly lower than those in the AN3CA/NC group (Figure 5E–5G). Xenograft immunohistochemistry revealed decreased expression of KDM4A and ki67 and increased expression of p27^kip1^ and H3K4me3 (Figure 5H). Western blots confirmed that KDM4A expression in both AN3CA and HEC-1B cells was higher than found in normal endometrial cells (Supplementary Figure 2A and 2C).

### Clinicopatholical significance of KDM4B and KDM4A expression in EC

**Figure 7:**
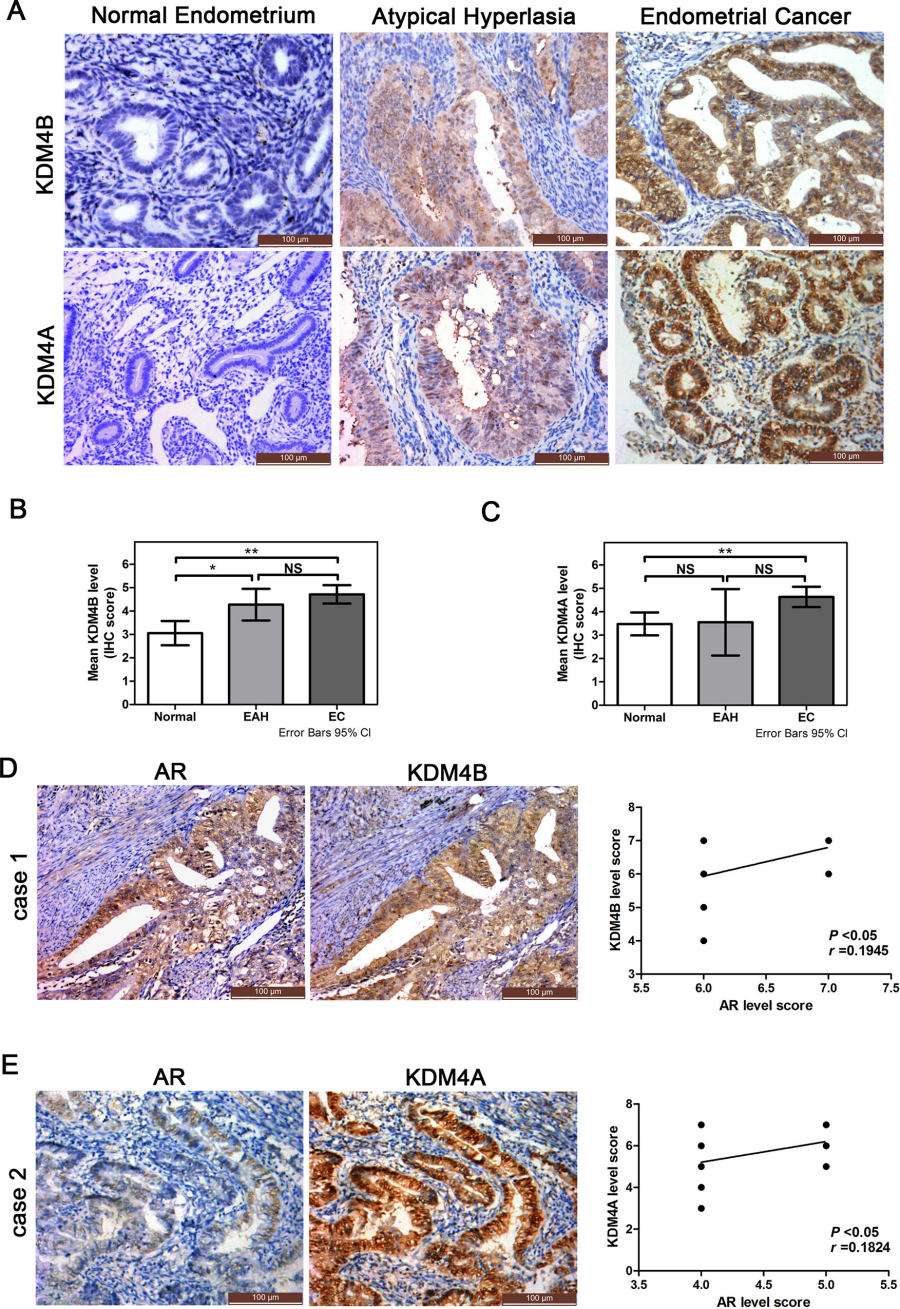
Expression of KDM4B and KDM4A in endometrial tissues and their relationship to AR expression in EC specimens **A.** Immunohistochemical stain of KDM4B and KDM4A in normal endometrium, endometrial atypical hyperplasia (EAH), and endometrioidcancer (EC) (200×). Summary of the immunostaining scores of **B.** KDM4B and **C.** KDM4A in normal endometrium, EAH and EC (*P < 0.05, **P < 0.01; NS, not significant). A score of ≥4 was considered positive for KDM4B and KDM4A expression. **D.** Example of KDM4B and AR immunoreactivity in EC tissues. KDM4B and AR levels were correlated in the 21 ECs with high AR scores (6 or 7) (*r* = 0.1945, *P* < 0.05). **E.** Example of KDM4A and AR immunoreactivity in EC tissues. KDM4A and AR levels were correlated in the 29 ECs with lower AR scores (4 or 5) (*r* = 0.1824, *P* < 0.05).

Given the roles of KDM4B and KDM4A in EC cell AR signaling, expression of these two enzymes was assessed in human EC clinical material (Figure 7A). In total, 57 normal endometrium, 11 atypical hyperplasia, and 76 EC cases were included in this study; we had assessed AR expression in these tissues previously [7]. KDM4B and KDM4A levels were higher in EC than in atypical hyperplasia, which had higher levels than normal endometrial tissues (*P < 0.05, **P < 0.01, Figure 7B and 7C). Furthermore, higher levels of KDM4B correlated with advanced tumor grade and depth of myometrial invasion in clinical EC specimens (*P < 0.05, Table 1). The 50 cases of EC that had expressed AR in our previous study were separated into two groups based on AR levels: tissues with an IHC score of 6 or 7 were included in the high-AR group, and those with an IHC score of 4 or 5 were included in the low-AR group [7]. KDM4B staining was positively correlated with AR expression in the high-AR group (Figure 7D), whereas KDM4A staining was positively correlated with AR expression in the low-AR group (Figure 7E).

**Table 1:** Relationship between KDM4B/KDM4A expression and clinicopathological features in 76 endometrial cancer tissue samples

Parameter	No.	KDM4B		*p*	KDM4A		*p*
		High	Low		High	Low	
Age (years)							
≤55	31	19	12	0.183	20	11	0.841
>55	45	34	11		28	17	
FIGO stage							
I	58	39	19	0.708	36	22	0.885
II	6	4	2		4	2	
III	11	9	2		7	4	
IV	1	1	0		1	0	
Pathological type							
Endometrioid	63	43	20	0.743	40	23	1.000
Non-endometrioid (serous/clear)	13	10	3		8	5	
Histological grade							
G1	35	19	16	0.029	24	11	0.593
G2	22	19	3		13	9	
G3	6	5	1		3	3	
Lymph node metastasis							
Positive	10	7	3	1.000	8	2	0.308
Negative	66	46	20		40	26	
Depth of myometrial invasion							
≤1/2	47	28	19	0.020	27	20	0.227
>1/2	29	25	4		21	8	
ER_α_ expression							
Positive	61	43	18	1.000	39	22	0.999
Negative	15	10	5		9	6	
P53 expression							
Positive	20	15	5	0.588	11	9	0.425
Negative	56	38	18		37	19	

**Figure 8:**
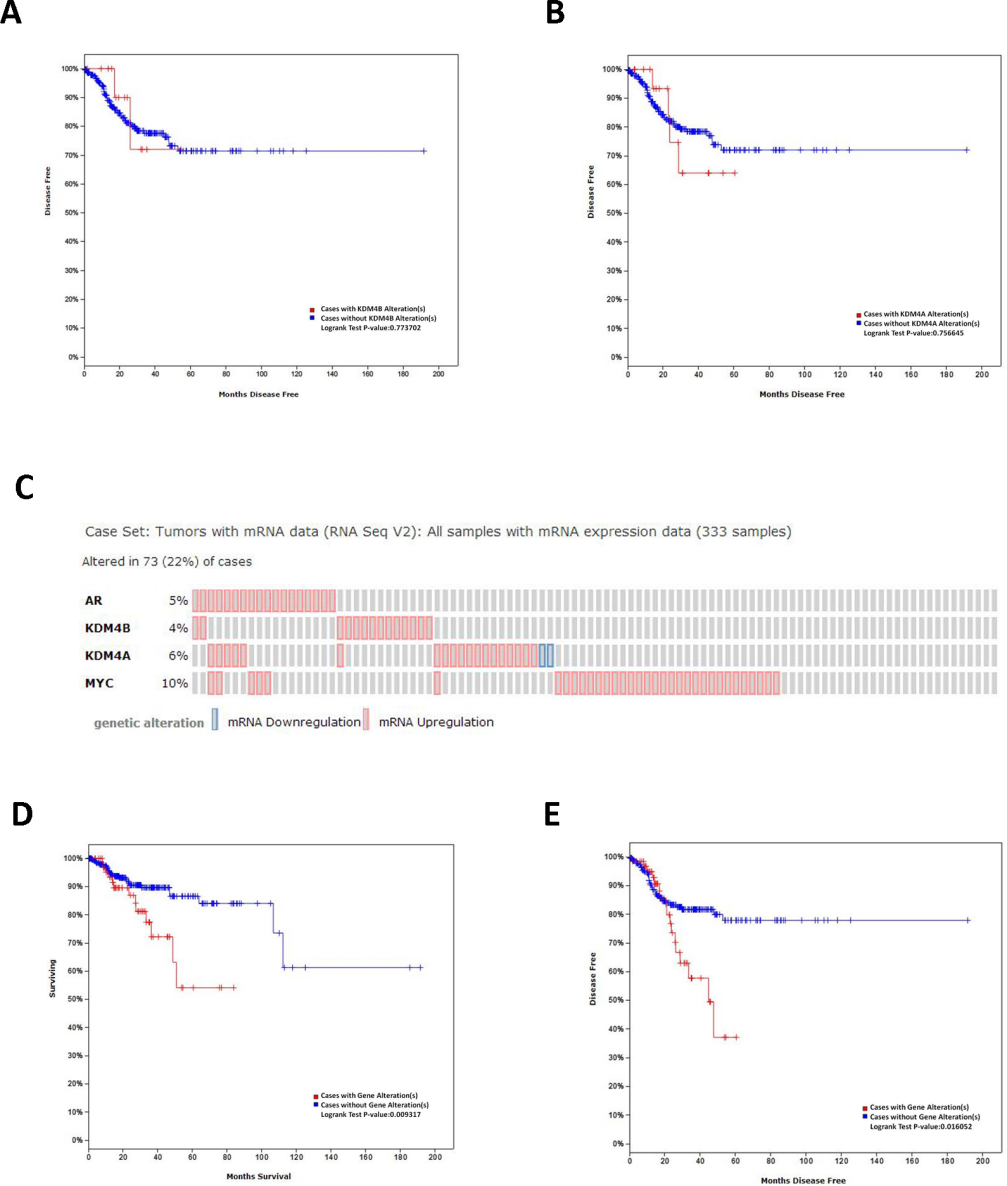
*In silico* analysis of OS and DFS in EC patients The Kaplan-Meier disease-free survival (DFS) of uterine corpus endometrioid carcinoma patients (*n* = 333) from a dataset in The Cancer Genome Atlas stratified by **A.** KDM4B and **B.** KDM4A expression. **C.** OncoPrint of AR, KDM4B, KDM4A, c-myc pathway alterations in EC. Genomic alteration provides an overview of genomic alterations (legend) in particular genes (rows) affecting particular individual sample (columns). The Kaplan-Meier **D.** overall survival (OS) and **E.** DFS of uterine corpus endometrioid carcinoma patients from a dataset in The Cancer Genome Atlas stratified by AR, KDM4B, KDM4A, and c-myc alteration.

cBioPortal was used to identify relationships between KDM4B and KDM4A expression and disease-free survival (DFS) of uterine corpus endometrioid carcinoma (UCEC) patients (*n* = 333) in an independent dataset from TCGA network. Although UCEC patients with KDM4B and KDM4A alteration seemed to have poorer DFS rates, this effect did not reach statistical difference (*P* > 0.05, Figure 8A and 8B). However, after further analysis of KDM4B, KDM4A, AR, and c-myc in this independent dataset, we discovered that 73 of 333 patients (22%) had an alteration, accompanied by mRNA up-regulation, in at least one of these genes (Figure 8C). Alterations of these genes were associated with poor overall survival (OS) and DFS in UCEC patients (*P* < 0.05, Figure 8D and 8E).

## DISCUSSION

**Figure 9:**
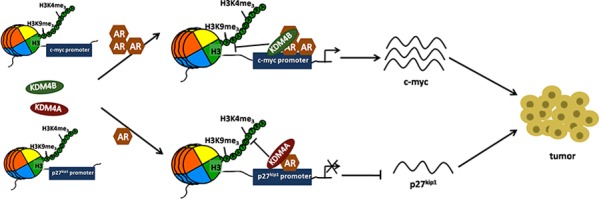
Schematic of KDM4B and KDM4A regulation of AR signaling in EC In EC tissues with high levels of AR, KDM4B, along with AR, is recruited to cis-regulatory elements of AR-target gene c-myc and activates c-myc expression by H3K9me3 demethylation. In EC tissues with low level of AR, KDM4A, along with AR, is recruited to cis-regulatory elements of AR-target gene p27^kip1^ and decreases expression of this tumor suppressor by H3K4me3 demethylation. These alterations in c-myc and p27^kip1^ expression promote EC progression.

In this study, we identified a novel AR-KDM4 (KDM4B/KDM4A) signaling axis that regulates AR target genes (c-myc/p27^kip1^) and accelerates EC progression (Figure 9 and Supplementary Figure 5). In addition, we describe a comprehensive mechanism of EC tumorigenesis, in which two different KDM4 family members promote EC progression by either upregulating or downregulating AR activity in different EC subtypes. Our results suggest that the AR-KDM4B pathway may serve as a novel therapeutic target for EC cells with high AR expression, whereas the AR-KDM4A pathway may serve as a novel therapeutic target for EC cells with low AR expression. The information gained from this research has important clinical implications for patients with EC and possibly for patients with other diseases associated with abnormal AR signaling.

The effects of AR activity on disease progression vary depending on cancer type. Elevated expression of AR has been associated with poor survival in prostate cancer and ERα-negative breast cancer patients [13, 19]. However, increased expression of AR in bladder cancer and ERα-positive breast cancer can be a good prognostic indicator [20, 21]. The role of AR in EC remains unclear. In one study, AR inhibited the growth of endometrial epithelial cells in culture, and an AR antagonist blocked this effect [5]. In another study, AR increased CD133 expression, cell migration, and epithelial-mesenchymal transition [6].

We previously found that AR increases EC cell proliferation, at least partly by activating oncogene c-myc [7]. Here, we demonstrated that KDM4 family member KDM4B promotes EC progression by activating the AR signaling cascade in MFE-296 cells, which have high AR levels. KDM4B and AR are recruited to cis-regulatory elements of AR, target gene c-myc, activating c-myc expression by H3K9me3 demethylation. C-myc is classified as a proto-oncogenic transcription factor, and abnormal activation of c-myc is found in many types of cancer, including EC [22–24]. C-myc promotes tumor growth through AR signaling in a variety of AR-related cancers, such as prostate, breast, and bladder cancers [25–27]. However, the AR-c-myc cascade in EC has not been fully investigated. Our data indicate that KDM4B-dependent epigenetic modifications upregulate the AR-c-myc cascade and promote EC, at least in MFE-296 cells.

Intriguingly, we also discovered that KDM4A, another KDM4 family member, suppresses the AR signaling cascade in AN3CA cells, which have relatively low AR levels. However, KDM4A also promotes EC progression. KDM4A and AR are recruited to cis-regulatory elements of the AR target gene p27^kip1^, where they inhibit the expression of that tumor suppressor via H3K4me3 demethylation. This inhibition of p27^kip1^ increased AN3CA cell proliferation and metastasis. Other proteins that suppress p27^kip1^, such as Skp2, also promote EC cell proliferation [28]. Our results provide evidence for another distinct anti-tumorigenic role of p27^kip1^ and highlight the importance of KDM4A expression in EC.

Although it is interesting that two different KDM4 proteins act via two distinct mechanisms to promote EC in both low and high AR cell lines, similar effects have been observed in other cancers. For example, KDM4B promotes prostate cancer carcinogenesis via the alteration of AR signaling [13], and the AR-KDM4A complex promotes prostate cancer progression [11]. To further complicate matters, KDM4A can also suppress tumor growth by coregulating AR signaling in bladder cancer [29]. A single KDM4B methylase, then, can have opposite effects on AR signaling and disease progression in different types of cancer. Our findings that the role of KDM4 proteins can also vary in a single cancer depending on cell type adds even more complexity to the interpretation of KDM4 proteins’ role in cancer. Regardless, dysregulation of KDM4B and KDM4A levels has been reported in several types of solid tumors [13, 30, 31] in addition to EC, and the signaling mechanisms involved merit further study.

Our *in silico* analysis using the TCGA database confirmed that combined expression of AR, KDM4A, KDM4B and c-myc is predictive of EC survival. However, levels of c-myc alone had even better predictive values (Supplementary Figure 6). There are several potential explanations for this observation. The number of samples from patients with advanced FIGO stages and lymph node metastasis in TCGA database is limited, which may contribute to the lower predictive value of the combined expression levels. Additionally, expression of these proteins may differ depending on the population examined (IHC assay showed more KDM4 family alternations in the Chinese population as compared to the US). Finally, the tumor suppressing activity of AR in some EC cell lines and studies might limit its usefulness as a predictor of outcome [32]. Further investigation is required to test whether combined expression of AR, KDM4B, KDM4A, c-myc and p27^kip1^ can provide an improved predictive value.

Additional studies are necessary to clarify the roles of KDM4 epigenetic regulators, AR, and downstream target genes in EC and in other types of cancer. Especially of links between epigenetics and cancer in general are necessary; indeed, hormone receptors themselves also affect carcinogenesis through histone modifications [33]. Future immunohistochemical studies comparing AR and KDM4 family expression might help identify differences between normal endometrium and different EC tissue subtypes. Additionally, a long term (more than 5 years) follow-up study of the patients who provided the immunohistochemical data might help determine the impact of KDM4B and KDM4A levels on EC prognosis. Ultimately, such studies might aid in the development of therapies that differentially target AR and KDM4 family members depending on EC subtype.

## MATERIALS AND METHODS

### Ethics statement

This study was approved by the Human Investigation Ethical Committee of International Peace Maternity & Child Health Hospital affiliated with the Shanghai Jiao Tong University School of Medicine. The samples of EC, atypical hyperplasia and normal endometrial tissues were collected after obtaining written informed consent from the patients. The animal research was carried out in strict accordance with the recommendations in the Guideline for the Care and Use of Laboratory Animals of China. The protocol was approved by the Committee on the Ethics of Animal Experiments of the Obstetrical and Gynecological Hospital affiliated with Fu Dan University (permit number SYXK (hu) 2008–0064). All efforts were made to minimize animal suffering.

### Human tissue collection

Tissue samples from 76 cases of uterine EC were obtained from patients who underwent surgery between June 1, 2011 and April 30, 2013 at the International Peace Maternity & Child Health Hospital affiliated with the Shanghai Jiao Tong University School of Medicine. The stages and histological grades of these tumors were established according to the criteria of the Federation International of Gynecology and Obstetrics (FIGO) surgical staging system (2009) [34]. Two independent pathologists verified the histological diagnosis of all the collected tissues. The features of all EC tissue samples are provided in Table 1. A total of 57 tissue samples of normal endometrium and 11 samples of atypical hyperplasia were obtained from patients who had undergone hysterectomies to treat other diseases, such as myoma or adenomyosis. None of the patients had undergone hormone therapy, radiotherapy, or chemotherapy before surgery.

### Cell culture and transient transfections

Human EC cell lines MFE-296, KLE, HEC-1B, and AN3CA were purchased from the Chinese Academy of Sciences Committee Type Culture Collection cell bank. MFE-296 cells are derived from a moderately-differentiated primary endometrial adenocarcinoma (grade 2) with high level of AR [35], while AN3CA cells are derived from a metastatic endometrial adenocarcinoma (grade 3) with low level of AR [36]. KLE, HEC-1B, and AN3CA EC cell lines were grown in Dulbecco's modified Eagle's medium (DMEM)/F12 (HyClone, Waltham, MA, USA), whereas MFE-296 cell lines were grown in high-glucose DMEM (4.5 g/L glucose) supplemented with 10% fetal bovine serum (Gibco, Carlsbad, CA, USA) in a humidified atmosphere of 5% CO_2_ at 37°C.

Cell transient transfections were performed as we previously reported [7]. In brief, cells were seeded at 2 × 10^5^ cells/well in 6-well plates. After the cells were grown overnight, they were transfected with JMJD1C(siJMJD1C) (sc-75357, Santa Cruz), siKDM5A(sc-96023, Santa Cruz), siKDM4A, siKDM4B, siKDM4C, siKDM4D, siSMAD4, siKDM1A, siAR, or siRNA negative control (siNC) (all from GenePharma, Shanghai, China) using Lipofectamine^2000^ (Invitrogen, Carlsbad, CA, USA) according to the manufacturer's instructions. The sequences are listed in Table 2. The plasmid PWP1/GFP/Neo-AR containing transfection-ready AR cDNA (exAR) and its negative control PWP1/GFP/Neo were gifts from Doctor Yuyang Zhao at Shanghai First People's Hospital. The plasmid pCMV-HA-KDM4A containing transfection-ready KDM4A cDNA (Plasmid #24180), its negative control pCMV-HA (Plasmid #32530), pcDNA3/HA-FLAG-KDM5A containing trasfection-ready KDM5A cDNA (Plasmid #14800), and its negative control pcDNA3/HA-FLAG (Plasmid #10792) were purchased from Addgene (Cambridge, MA, USA). Cells were transfected with the specific plasmid using Lipofectamine^2000^ (Invitrogen) according to the manufacturer's instructions.

**Table 2:** siRNA and shRNA sequences used in the study

siRNA name	Primer sequences (5′-3′)
siKDM4A-F	GAGUUAUCAACUCAAGAUA
siKDM4A-R	UAUCUUGAGUUGAUAACUC
siKDM4B-F	CGGCCACAUUACCCUCCAA
siKDM4B-R	UUGGAGGGUAAUGUGGCCG
siKDM4C-F	GGCCUUAUGUGGUGAACAUTT
siKDM4C-R	AUGUUCACCACAUAAGGCCTT
siKDM4D-F	GAGAGACCUAUGAUAAUAUTT
siKDM4D-R	AUAUUAUCAUAGGUCUCUCTT
siSMAD4-F	GGUGGAGAGAGUGAAACAUTT
siSMAD4-R	AUGUUUCACUCUCUCCACCTT
siKDM1A-F	CCGGAUGACUUCUCAAGAATT
siKDM1A-R	UUCUUGAGAAGUCAUCCGGTT
siAR-F	AUGUCAACUCCAGGAUGCUTT
siAR-R	AGCAUCCUGGAGUUGACAUTT
siNC-F	UUCUCCGAACGUGUCACGUTT
siNC-R	ACGUGACACGUUCGGAGAATT
shKDM4B	AAACCCAGGGCTGCCTTGGAAAAG
shKDM4A	AAACCCAGGGCTGCCTTGGAAAAG

### Primary cell culture from normal endometrium

Tissue specimens of normal endometrial cells were obtained from ten women with endometriosis who had given informed consent before surgery. These women had no endometrial hyperplasia or neoplasia, and had not received any hormonal medication or anti-inflammatory drugs for at least 3 months prior to surgery. The endometrial biopsies were minced into small pieces and digested with 0.2% collagenase (Gibco). Cells were pelleted by centrifugation, resuspended in Dulbecco's modified Eagle's medium (DMEM)/F12 containing 50 U/ml penicillin, 50 μg/ml streptomycin, and 10% FBS, and plated in 100-mm diameter culture dishes, then grown at 37°C in 5% CO_2_ [37]. Epithelial cells were separated from stromal fibroblast-like cells morphologically in culture by light microscopy; epithelial cells formed colonies that spread out gradually and exhibited a large and polygonal morphology (Supplementary Figure 2A). Epithelial cells were also identified immunocytochemically with specific monoclonal antibodies to cytokeratins and vimentin [38].

### 5α-Dihydrotestosterone treatment

Cells were incubated in phenol red–free DMEM/F12 (Gibco) overnight, with the final concentration of ethanol at 0.1% 5α-dihydrotestosterone (DHT) (Dr. EhrenstorferGmbh, Augsburg, Germany) was added to the cell culture media at concentrations of 10^−7^ M for different periods of time (0–24 h). Vehicle contained 0.1% absolute ethanol/phenol red–free DMEM/F12.

To investigate the impact of KDM4A and KDM4B combined with DHT treatment, cells that had been transfected with siKDM4A, siKDM4B, or siNC for 36 h were incubated with phenol red–free DMEM/F12 for 12 h. DHT or vehicle was then added according to the methods mentioned above. Previous dose-response experiments revealed that 10^−7^ M (100 nM) DHT and 24 h of incubation elicited the strongest expression of AR and its target genes [7].

### Quantitative real-time polymerase chain reaction

Total RNA was extracted from cultured cells using Trizol Reagent (Invitrogen). RNA was converted to cDNA with a one-step Prime Script RT reagent kit (TaKaRa, Dalian, China), and quantitative real-time polymerase chain reaction (PCR) was performed by using SYBR Premix Ex Taq (TaKaRa) in an Eppendorf Mastercycler^®^ realplex according to the manufacturer's instructions. Each sample was assayed in triplicate in each of three independent experiments. The primer sequences are listed in Table 3.

**Table 3:** Primers used for qRT-PCR and ChIP-PCR

mRNA	Primer sequence
KDM4B	Forward 5′-CCAGAGGCTTCCTTGCAGACAA-3′
	Reverse 5′-CCAAACTCCTGCCTCAGCCATT-3′
c-myc	Forward 5′-AAAGGCCCCCAAGGTAGTTA-3′
	Reverse 5′-TTTCCGCAACAAGTCCTCTT-3′
XBP1	Forward 5′-CCTTGTAGTTGAGAACCAGG-3′
	Reverse 5′-GGGGCTTGGTATATATGTGG-3′
KDM4A	Forward 5′-GGAAGCCACGAGCATCCTATG-3′
	Reverse 5′-GGAACTCTCGAACAGTCATGG-3′
P27^kip1^	Forward 5′-AAGCAACAGAAACCTATCCTCA-3′
	Reverse 5′-TTTACACAGCCCGAAGTGAA-3′
c-myc promoter	Forward 5′-CCCCCGAATTGTTTTCTCTT-3′
	Reverse 5′-TCTCATCCTTGGTCCCTCAC-3′
P27^kip1^ promoter	Forward 5′-CTGTCACATTCTGGAGCGTA-3′
	Reverse 5′-AGTGGATCTTCAACTGCCTC-3′

**Abbreviations:** ChIP-PCR, Chromatin immunoprecipitation-polymerase chain reaction; qRT-PCR, quantitative real-time polymerase chain reaction

### Western blotting

For Western blot assays, conducted as previously described [7], antibodies against the following proteins were used: KDM4A (Novus, Littleton, CO, USA, NB110–40585), KDM4B (Bethyl Laboratories, Montgomery, TX, USA, A301–478A), KDM4C (Novus, NBP1–49600), KDM4D (Abcam, Hong Kong, China, ab198209), AR (Santa Cruz, sc-7305), histone H3 trimethyllysine 9 (H3K9me3) (Abcam, ab8898), histone H3 trimethyl lysine 4 (H3K4me3) (Abcam, ab8580), H3 (Abcam, ab1791), c-myc (Santa Cruz, sc-789), and p27^kip1^ (Santa Cruz, sc-508). Protein bands were visualized by enhanced chemiluminescence (Pierce Biotechnology, Rockford, IL, USA), and protein expression was normalized against β-actin (Cell Signaling Technology, Danvers, MA, USA).

### Co-immunoprecipitation

Total protein was extracted from MFE-296 cells. After protein quantification, 500 μg of each cell lysate was added to 10 μl of AR or KDM4B antibody, shaken at 4°C overnight, and then added to 30 μl of Protein A + G Agarose (Beyotime Institute of Biotechnology, Haimen, Jiangsu, China), shaken at 4°C for 4 h, centrifuged at 2500 × g for 5 min, and washed with a RIPA kit (Beyotime) to collect the immunoprecipitate-bound agarose beads. Each immunoprecipitate was denatured with 20 μl of 1× SDS-PAGE loading buffer at 100°C for 5 min. Each supernatant was subjected to SDS-PAGE (8% acrylamide). Other steps were as described in the Western blotting section.

To determine whether KDM4A or KDM4B can interact with AR in AN3CA cells, AN3CA cells grown in DMEM/F12 media containing 10% fetal calf serum were transfected with 1 mg of PWP1/GFP/Neo-AR or 1 mg of its negative control PWP1/GFP/Neo after 72 h using Lipofectamine^2000^ according to the manufacturer's protocol. Cells were harvested and subjected to immunoprecipitation using AR antibody (Santa Cruz), followed by Western blotting with KDM4B antibody (Bethyl Laboratories) and KDM4A antibody.

### Chromatin immunoprecipitation (ChIP)-PCR

Chromatin immunoprecipitation (ChIP) assays were performed as previously described [7, 39] using the antibodies for AR, KDM4A, KDM4B, KDM5A (Abcam, ab70892), histone H3 trimethyl lysine 9 (H3K9me3), histone H3 trimethyl lysine 4 (H3K4me3) or rabbit isotype IgG (Cell Signaling Technology). AR-binding sites within the c-myc promoter were depicted previously [7]. Both JASPAR [40]and PROMO [41] predicted that p27^kip1^ promoter between − 1523~− 1294 includes the AR binding site. Enrichment was calculated by using the comparative Ct method. Primers used (including p27^kip1^ promoter targeting the range − 1523~− 1294) are shown in Table 3 and were analyzed for linearity range and efficiency to accurately evaluate occupancy (percentage of immunoprecipitation/input). IgG was used as a negative control.

### MTT and colony formation assays

In MTT assays, cells were plated at 3 × 10^3^ cells/well in 96-well plates. Then, 20 μl of 3-(4,5-dimethylthiazol-2-yl)-2,5-diphenyltetrazolium bromide (MTT, 5 mg/ml; Sigma, St. Louis, MO, USA) was added to each well. Wells were subsequently incubated at 37°C for 4 h. Absorbance was measured at 490 nm using a Spectra Max 190 microplate reader (BIO-RAD, Hercules, CA, USA). In colony formation assays, cells were seeded at 120 cells/well (MFE-296 cells) or 200 cells/well (AN3CA cells) in 6-well plates. After 14 days, cell colonies were stained with 0.1% crystal violet for 1 h and counted.

### Cell migration and invasion assays

Cells were plated serum-free at a density of 1 × 10^5^/well (for the migration assay) or 2 × 10^5^/well (for the invasion assay) in invasion chambers (8-μm pore size; BD Biosciences, San Jose, CA, USA) with or without matrigel coating for invasion or migration assays. Complete medium was added to the lower chamber as a chemoattractant. After 5 h (MFE-296) or 24 h (AN3CA) of incubation for the migration assay, or after 24 h (MFE-296) or 48 h (AN3CA) of incubation for the invasion assay, cells were fixed with 4% paraformaldehyde for 1 h. Cells on the apical side of each insert were removed by mechanical scraping. Cells that migrated to basal side of the membrane were stained with 0.5% crystal violet and counted at 200× magnification. Cells migration and invasion assays were repeated at least three times.

### Generation of stable shRNA cell lines for xenograft assays

The lentivirus vector down-expressing KDM4B was prepared for MFE-296 cells using shRNA targeting KDM4B (pLKO.1, TRCN0000018013; Open Biosystems, Lafayette, CO, USA) or a negative control vector (pLKO.1, Open Biosystems) according to the manufacturer's protocol. The lentivirus vector down-expressing KDM4A was prepared for AN3CA cells using a shRNA targeting KDM4A (pLKO.1, TRCN0000013493; Open Biosystems) or a negative control vector (pLKO.1, Open Biosystems) according to the manufacturer's protocol. shRNA lentiviral particles were treated in six-well plates in the presence of polybrene (6 μg/ml). Cells were treated with puromycin (2 μg/ml) to generate stable KDM4B and KDM4A knockdown clones, and several puromycin-resistant KDM4B and KDM4A knockdown clones were harvested by ring selection. The shRNA oligo sequences are provided in Table 2.

### Xenograft assays

For the xenograft experiments, 20 5-week-old female BALB/c nude mice (Chinese Academy of Sciences, Shanghai, China) were randomly divided into four groups. The mice were injected subcutaneously in the nape with 5 × 10^6^ cells of one of the following cell types: MFE-296/shKDM4B, MFE-296/shNC, AN3CA/shKDM4A, or AN3CA/shNC. Tumor size was monitored every 4 days by measuring the length and width with calipers, and tumor volumes were calculated with the formula: tumor volume (cm^3^) = (the longest diameter) × (the shortest diameter)^2^ × 0.5. The tumors were removed and weighed after 42 days (for the two MFE-296 groups) or after 49 days (for the two AN3CA groups).

### Immunohistochemical analysis

Paraffin-embedded normal endometrial, atypical hyperplasias, and EC tissue sections (4 μm) were processed for immunohistochemical analysis as previously described [7]. Briefly, after deparaffinization and dehydration, specimens were boiled in EDTA (PH8.0) to unmask antigens. Then specimens were blocked and incubated with primary antibody overnight at 4°C. The staining intensity was rated in the following manner: 0 (negative), 1 (weak), 2 (medium), or 3(strong). The percentage of positively stained cells was rated as follows: 0 (0%), 1 (1%–25%), 2 (26%–50%), 3 (51%–75%), or 4 (76%–100%), according to the percentage of the positively stained areas in relation to the whole tumor area. Then, immunoreactivity scores for each case were obtained by adding the values of the two parameters described above [42]. A score of ≥4 was considered positive for KDM4B and KDM4A expression. We divided all 50 cases of EC that had positive AR expression in our previous study [7] into two groups according to AR level (an IHC score of 6 or 7 was the high-AR group; an IHC score of 4 or 5 was the low-AR group). Levels of KDM4A and KDM4B expression were evaluated and then analyzed separately in the tissues that comprised the high- and low-AR groups in our previous study [7].

In addition, tissue specimens (4 μm) of mouse xenografts were routinely prepared and immunohistochemically analyzed by using the standard avidin-biotin techniques. KDM4B antibody, c-myc antibody (Santa Cruz, sc-789), KDM4A antibody, p27^kip1^ antibody (Santa Cruz, sc-508), and ki-67 antibody (Boster, BA2888) were used.

### Integrative analysis of complex cancer genomics with use of the cBioPortal

The cBioPortal for Cancer Genomics (http://cbioportal.org) provides a Web resource for exploring, visualizing, and analyzing multidimensional cancer genomics data. Specifically, because of interests in the gene set of AR, we entered AR gene symbols, and then a report of genomic data across all cancer types was presented in a concise graphical summary called a *cancer genomics of cross-cancer alteration summary* of AR. We then selected *endometrial carcinoma*, entered AR and KDM4B gene symbols, and a network view of the *AR* neighborhood in EC was shown. All genes were ranked by the frequency of genomic alteration within the EC study, and less frequently altered genes were automatically pruned from the network.

### *In silico* analysis of KDM4A, KDM4B, AR, c-myc, and overall survival/disease-free survival of EC patients

We performed an *in silico* analysis of the association between KDM4A/KDM4B and overall survival (OS)/disease-free survival (DFS) of EC patients using published data from The Cancer Genome Atlas (TCGA) network [43, 44]. Clinical information and mRNA expression profiling by RNA-seq was downloaded from database. Differences in OS and DFS were computed between tumor samples that had at least one alteration in the genes of our interest. The results are displayed Kaplan-Meier plot with *P* values from a log-rank test.

### Statistical analysis

Data were presented as mean ± SD and analyzed by SPSS 16.0 software (SPSS Inc., Chicago, IL). The Student *t* test was used for comparison between two groups, and one-way ANOVA followed by the post-hoc Tukey test were used for comparison among multiple groups. Correlation was analyzed by the Spearman test, and OS was assessed by using the standard log-rank test and the Kaplan-Meier method. All *P* values were two-tailed, and *P* values < 0.05 indicated a statistically significant difference. Each experiment was performed at least three times independently.

## SUPPLEMENTARY FIGURES


