# Supplementary material for: KDM4B and KDM4A promote endometrial cancer progression by regulating androgen receptor, c-myc, and p27kip1
Source: Oncotarget. 2015 Sep 11;6(31):31702–20. doi: 10.18632/oncotarget.5165 (PMC4741634; doi:10.18632/oncotarget.5165)
Supplement: Supplementary file 1 [file oncotarget-06-31702-s001.pdf]

SUPPLEMENTARY FIGURES

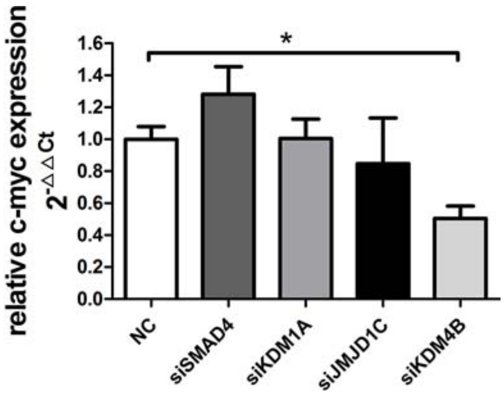

Supplementary Figure S1: Silencing of KDM4B down-regulated c-myc mRNA expression. Silencing of KDM4B down-regulated c-myc mRNA expression in qRT-PCR assays in MFE-296 cells (*P < 0.05), where as other candidate regulators (KDM1A, JMJD1C and SMAD4) did not affect c-myc expression (P > 0.05). All experiments were performed three times, and the data represent the mean fold change ± Standard Error (SE).

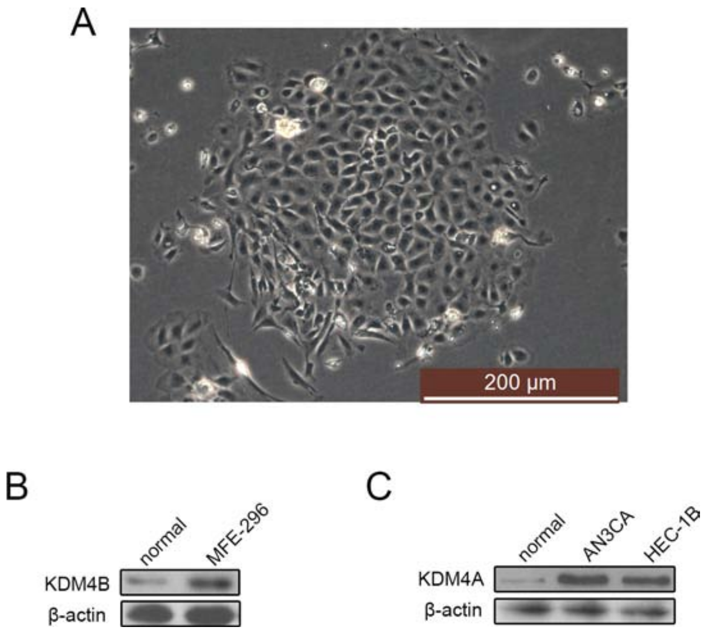

Supplementary Figure S2: Comparison of expression of KDM4B and KDM4A in normal endometrial cells to EC cells. A. Normal endometrial epithelial cells extracted from normal endometrial tissues in light microscopy. Magnification, 100×. **B.** A western blot evaluating KDM4B protein level in MFE-296 cells and normal endometrial cells extracted from normal endometrial tissue. **C.** A western blot evaluating KDM4A protein level in AN3CA cells, HEC-1B cells and normal endometrial cells extracted from normal endometrial tissue. β-actin was used as loading controls. All experiments were performed three times.

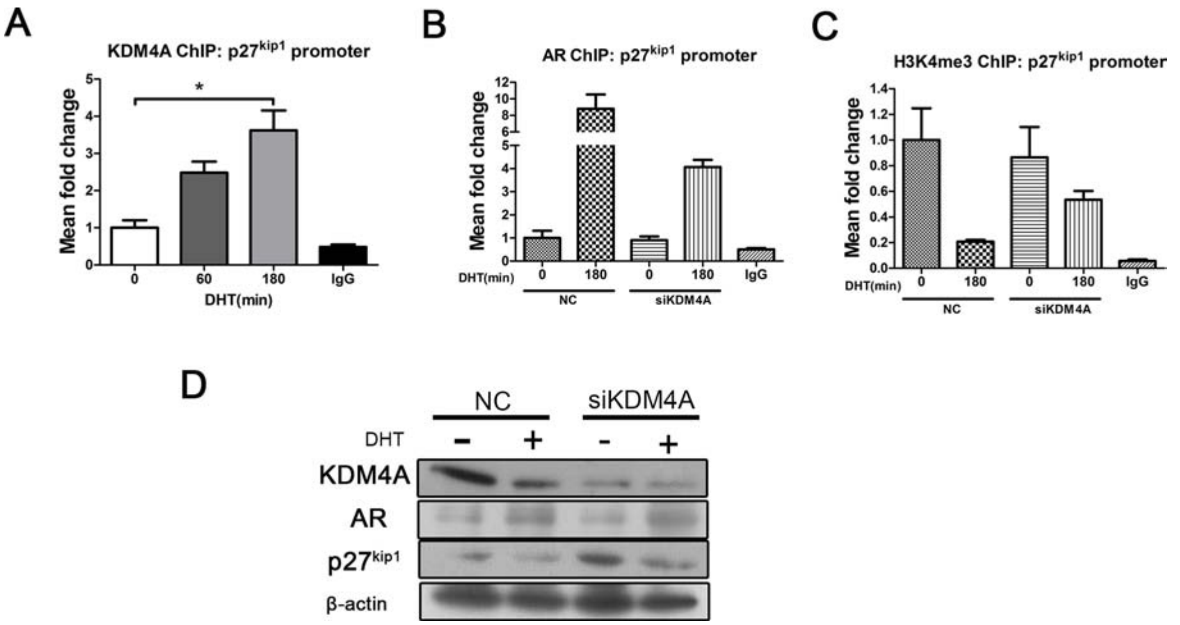

Supplementary Figure S3: KDM4A suppresses AR target $p27^{kip1}$ by demethylating H3K4me3 in response to androgens in HEC-1B cells. A series of ChIP for **A.** KDM4A at the $p27^{kip1}$ promoter in HEC-1B cells treated with DHT (100 nM) for 0, 60, and 180 min. *$P < 0.05$, compared with 0 min. **B.** HEC-1B cells were transiently transfected with either negative control (NC) or KDM4A siRNA (siKDM4A) in steroid-depleted media for 72 h with or without 180 min 100 nM DHT stimulation before ChIP with (B) an anti-AR antibody, or **C.** an anti- H3K4me3 antibody, and subsequently analyzed at the $p27^{kip1}$ promoter. Nonspecific IgG was used as a negative control. ChIP data are an average of three independent experiments ± SE. **D.** HEC-1B cells were subject to transient transfection with either negative control or KDM4A (siKDM4A) siRNAs in steroid-depleted media for 48 h before 24 h 100 nM DHT stimulation, and subsequently protein extraction. AR, KDM4A, $p27^{kip1}$were assessed by Western blotting, with β-actin as loading controls.

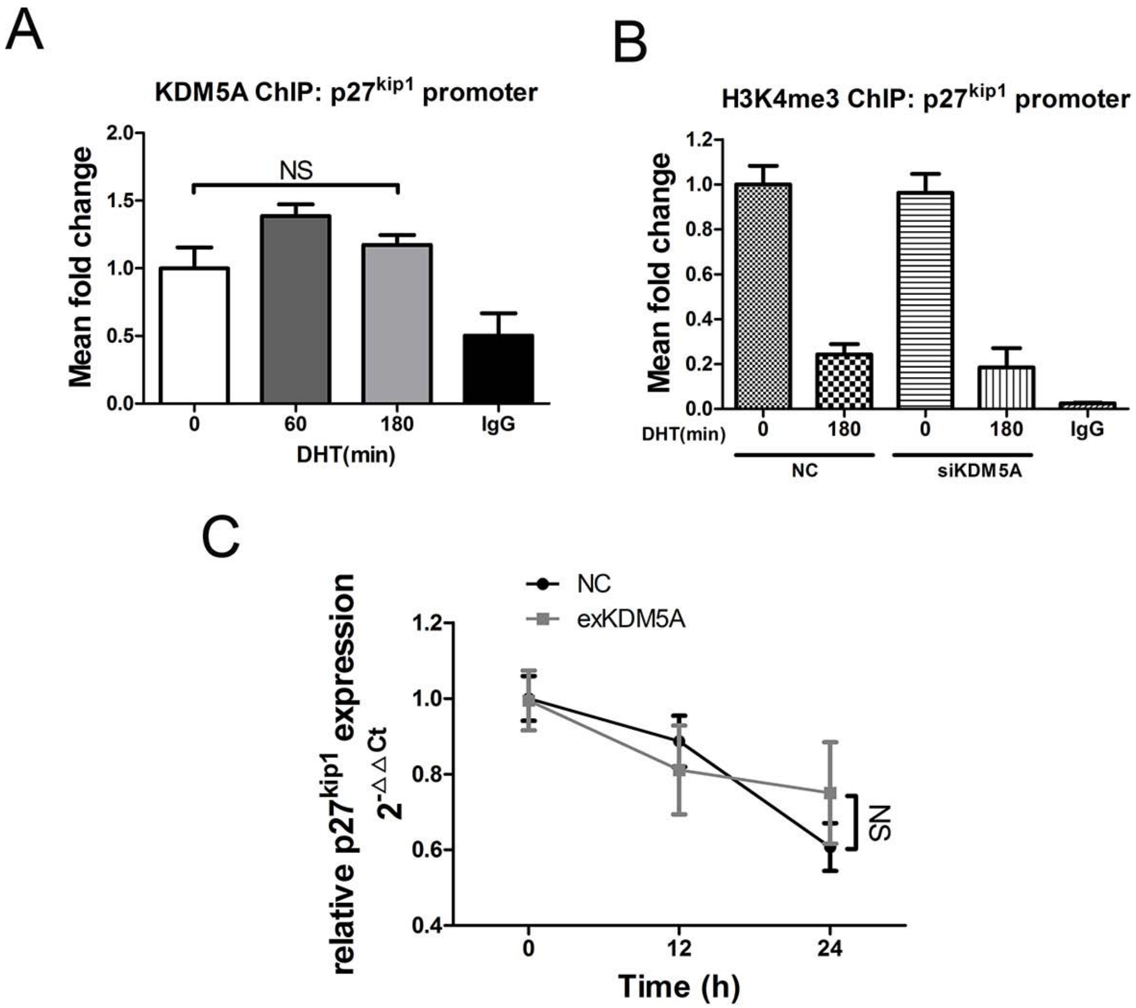

Supplementary Figure S4: KDM5A did not demethylate H3K4me3 and did not regulate p27^{kip1} in response to androgens in AN3CA cells. A series of ChIP for **A.** KDM5A at the p27^{kip1} promoter in AN3CA cells treated with DHT (100 nM) for 0, 60, and 180 min. NS, not significant. **B.** AN3CA cells were transiently transfected with either negative control (NC) or KDM5A siRNA (siKDM5A) in steroid-depleted media for 72 h with or without 180 min 100 nM DHT stimulation before ChIP with an anti-H3K4me3 antibody, and subsequently analyzed at the p27^{kip1} promoter. Nonspecific IgG was used as a negative control. ChIP data are an average of three independent experiments ± SE. **C.** AN3CA cells were transiently transfected with either negative control (NC) or KDM5A expression plasmid (exKDM5A). Overexpression of KDM5A had no obvious effect on regulating p27^{kip1} mRNA expression in qRT-PCR assays in AN3CA cells.

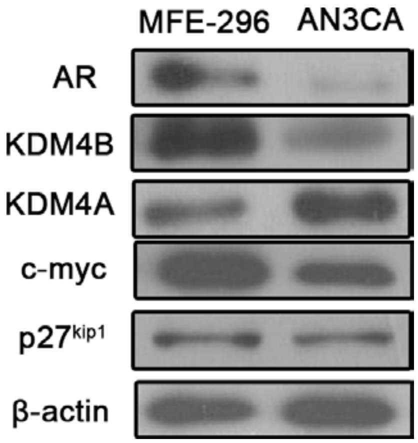

Supplementary Figure S5: Expression of AR, KDM4B, KDM4A, c-myc, and p27^{kip1} in MFE-296 and AN3CA cells. A western blot evaluating the expression of AR, KDM4B, KDM4A, c-myc, and p27^{kip1} in MFE-296 and AN3CA cells were shown. β-actin was used as loading controls.

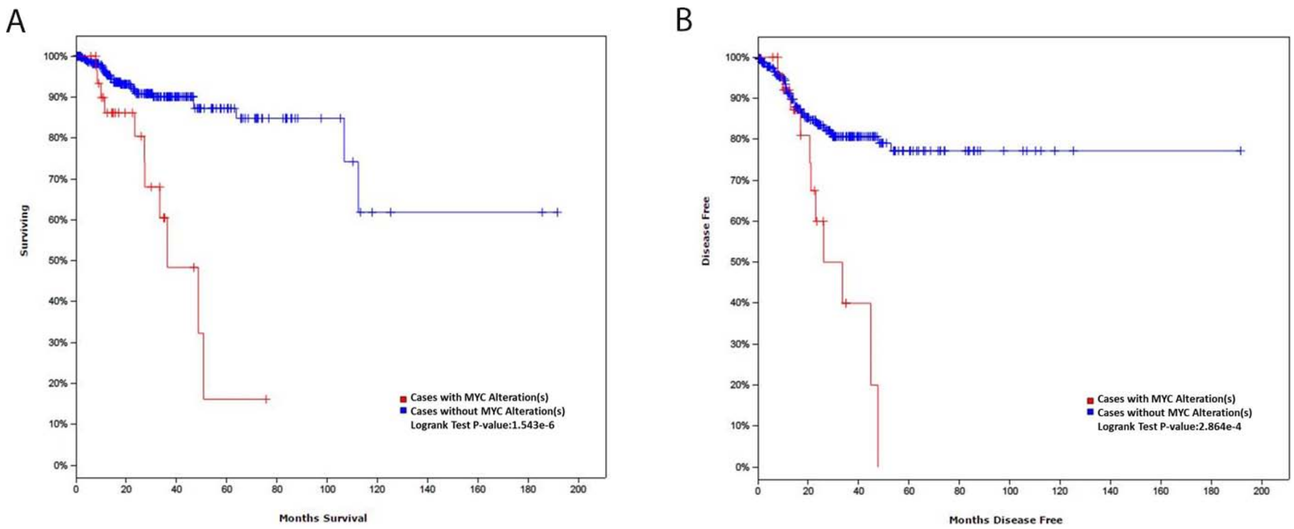

Supplementary Figure S6: *In silico* analysis identified OS and DFS in EC patients with c-myc alteration. The Kaplan-Meier **A.** overall survival (OS) and **B.** DFS of uterine corpus endometrioid carcinoma patients from a dataset in The Cancer Genome Atlas stratified by c-myc alteration.
